# Editorial: Age-Related Neuroimmunology of Degeneration and Repair

**DOI:** 10.3389/fnagi.2021.742620

**Published:** 2021-08-20

**Authors:** Khalil S. Rawji, Deepak K. Kaushik

**Affiliations:** ^1^Wellcome - MRC Cambridge Stem Cell Institute, Jeffrey Cheah Biomedical Campus, University of Cambridge, Cambridge, United Kingdom; ^2^Department of Clinical Neurosciences, Hotchkiss Brain Institute, University of Calgary, Calgary, AB, Canada; ^3^Division of Biomedical Sciences, Faculty of Medicine, Memorial University of Newfoundland, St. John's, NL, Canada

**Keywords:** neuroimmunology, aging, neurodegeneration, therapy, peripheral nervous system, central nervous system, immune activation

Our understanding of the cellular, molecular, and physiological components of an aging brain has increased over the last few decades, yet the role of the immune system in governing the repair and/or degeneration of the CNS during aging is less explored. It is critical to understand the functions of different immune cells involved in aged brains, with specific interests in degenerative conditions of both the central nervous system (CNS) as well as the peripheral nervous system (PNS). With this in mind, we launched the topic, “Age-Related Neuroimmunology of Degeneration and Repair,” in October 2019 with an aim to enhance our understanding of the role of the immune system in aging brains. Despite COVID-19-related delays, we are glad that the topic was well-received, which is showcased in publication of high-quality papers within the topic as this remains one of the most viewed compilations on Frontiers' platform today. The collection in this Research Topic span a broad variety of age-related conditions, including Parkinson's disease (Zhang et al., Lin C-H. et al., Chen et al.), Alzheimer's disease (AD) (Pollock et al., Yin et al., Han et al.), stroke (Wang et al.), cognitive decline (Jiang et al.), as well as conditions affecting the peripheral nervous system. The articles presented herein extend from basic science inquiries into the immunological properties underlying some of these conditions all the way to studies examining potential novel therapeutic compounds.

This Research Topic benefits from a series of comprehensive review articles, exploring several different facets of neuroinflammation in the context of aging and disease. Of particular note, Mayne et al. provide an extensive overview of the association between aging and several neurodegenerative diseases, including an analysis of the roles potentially played by the adaptive immune system, an arm of age-related neuroinflammation currently understudied. On another note, Han et al., discuss the critical role of astrocyte senescence in mediating AD pathology, a cell type that has garnered attention in recent years in regards to their immunological roles within the CNS. In addition, the role of gut microbiota did not go unnoticed, and dysbiosis in AD is the focus of the review by Zhu et al.. The collection was also enriched by the discussions on relevance of checkpoint inhibition, particularly in tau pathology, in a commentary by Baruch and Yoles and its response by Lin Y et al..

Another highlight of this Research Topic is the inclusion of two articles focusing on the aging peripheral nervous system, another area that has been considerably underappreciated in neuroimmunology. Stratton et al. perform a very nice study examining the immunological factors underlying the age-related impairment in peripheral nerve regeneration. In this study, they find that bone marrow transplantation from aged mice into young ones is sufficient to overcome the impairment in functional recovery in the peripheral nerve, suggesting that the young microenvironment plays a significant role in reversing any age-related intrinsic changes in the hematopoietic stem and progenitor cell populations. This study then shows through transcriptional profiling that the age-related impairment in peripheral nerve regeneration may be attributed to defects in monocyte chemoattractant protein-1 signaling. Hagen and Ousman provide an overview of another aspect of neuroimmunology of the peripheral nervous system, focusing on the autoimmune condition known as Guillain-Barré Syndrome. In this review, they summarize the various roles played by many different immune cells in this disease and present a conceptual framework looking into the potential impact an aging immune system has on this disease.

Overall, we hope that this Research Topic adds value to the existing literature and guides future research on the roles of the immune system in the age-related degeneration and repair of the central and peripheral nervous systems.

## Author Contributions

DKK and KSR contributed equally to the editorial.

## Conflict of Interest

The authors declare that the research was conducted in the absence of any commercial or financial relationships that could be construed as a potential conflict of interest.

## Publisher's Note

All claims expressed in this article are solely those of the authors and do not necessarily represent those of their affiliated organizations, or those of the publisher, the editors and the reviewers. Any product that may be evaluated in this article, or claim that may be made by its manufacturer, is not guaranteed or endorsed by the publisher.

